# Editorial: New Insights Into the Landscape of Rare Tumors: Translational and Clinical Research Perspective

**DOI:** 10.3389/fonc.2020.593785

**Published:** 2020-10-29

**Authors:** Alessandro De Vita, Alberto Bongiovanni, Jean-Yves Blay, Toni Ibrahim

**Affiliations:** ^1^ Osteoncology and Rare Tumors Center, Istituto Scientifico Romagnolo per lo Studio e la Cura dei Tumori (IRST) Istituto Di Ricovero e Cura a Carattere Scientifico (IRCCS), Meldola, Italy; ^2^ Department of Medical Oncology, Centre Léon Bérard, Lyon, France; ^3^ Unicancer, Paris, France; ^4^ EURACAN (European network for Rare adult solid Cancer) EC 739521, Lyon, France

**Keywords:** rare tumors, bone and soft tissue sarcoma, neuroendocrine neoplasms and endocrine gland tumors, central nervous system neoplasms, cholangiocarcinoma, von Hippel-Lindau disease

In the landscape of solid and liquid malignancies, rare tumors represent a major challenge for patient management in terms of their biology, classification, clinical behavior (1). In this scenario, the term *rare tumor* refers to a multitude of heterogeneous diseases often characterized by diagnostic pitfalls, the unavailability of prognostic and predictive biomarkers, and the lack of standardized treatments (2). Such issues are reflected in clinical outcome, which is generally worse than that of patients with more common tumors (3). Consequently, there is a pressing need for a better understanding of the natural history of these diseases and for the development of innovative treatment strategies (4, 5). Building a strong collaborative network between physicians, researchers, and institutions will be key to achieving the above goals (6).

The Research Topic of this special issue takes an in-depth look at recent translational and clinical advancements in the area of rare tumors. A collection of original articles, systematic reviews, methods, opinions and perspectives will provide readers with news on exciting breakthroughs in research into sporadic rare tumors including bone and soft tissue sarcoma (7, 8), neuroendocrine and endocrine gland neoplasms (9), brain tumors (10), cholangiocarcinoma (11), and rare familial syndromes such von Hippel-Lindau disease (12).

In this regard, the limited availability of biomarkers represents a substantial problem for the management of sarcomas. Furthermore, the unusual histologic features of these malignancies increase the risk of misdiagnosis, leading to the use of an ineffective therapeutic strategy and a poor outcome. Next-generation sequencing (NGS) technology would appear to be a promising tool for sarcoma diagnosis. The identification of histotype-specific gene alterations is of paramount importance for the differential diagnosis of sarcoma variants as almost 30% of sarcomas harbor specific genetic alterations. Racanelli et al. focused on NGS RNA-based approaches to detect sarcoma-specific rearrangements, confirming their potential usefulness in routine diagnostic setting. This is especially important as the identification of a specific genetic alteration may form the basis for a therapeutic option. For example, gastrointestinal stromal tumors (GIST) commonly harbor KIT or PDGFRA mutations and less frequently show SDH or NF1 gene inactivation. Only 10% of GIST are wild type, thus limiting therapeutic opportunities and increasing the risk of poor outcome. Astolfi et al. investigated the accuracy of NGS-based second-level molecular analysis for wild type GIST diagnosis. They found that 20% of wild type GIST harbored pathogenic KIT mutations and became eligible for TK inhibitors, underlining the importance of NGS technologies as a diagnostic tool.

The identification of prognostic biomarkers represents another unmet clinical need. Cheng et al. evaluated pretreatment inflammatory indexes as a prognostic predictor of survival in patients with synovial sarcoma, concluding that neutrophil-to-lymphocyte ratio (NLR) and lymphocyte-to-monocyte ratio (LMR) were independent prognostic factors of progression-free survival (PFS) and overall survival (OS) in this sarcoma histotype. An increasing interest is also being shown in hematological markers as a reliable prognostic tool for sarcoma management. Li et al. discussed the role of prognostic hematological biomarkers in soft tissue sarcoma. In particular, they found that higher NLR, C-reactive protein (CRP), and platelet-to-lymphocyte ratio (PLR) were associated with poor OS/disease-free survival (DFS), whereas a low LMR was linked to worse OS/DFS. Moreover, higher Glasgow prognostic scores (GPS) were correlated with poorer OS/disease-specific survival (DSS). Despite the discovery of genetic aberrations and a deeper understanding of their role in sarcoma pathophysiology, the cornerstone of treatment for the majority of these advanced and metastatic tumors, chemotherapy, has changed little over the years. Thus, the identification of predictive biomarkers to discriminate between responsive and non-responsive patients would represent an important step forward in the management of the disease. Caruso and Garofalo provided an overview of soft tissue sarcoma pharmacogenomic biomarkers currently used to monitor the responsiveness and toxicity of conventional and new chemotherapeutic drugs in soft tissue sarcoma histotypes. For example, extraskeletal myxoid chondrosarcoma is a rare soft tissue sarcoma characterized by an indolent behavior but with an increasing proportion of patients who develop local and distant recurrences. In the latter, standard front-line treatment using anthracycline-based chemotherapy has shown limited activity. Chiusole et al. retrospectively investigated a series of extraskeletal myxoid chondrosarcoma patients, observing that a primary tumor site in the extremities and solitary lung metastases were associated with a better survival. Their results also highlighted a poor performance of anthracycline-based chemotherapy, indicating the need to identify other active treatments.

Similar problems are encountered for the management of bone sarcoma, which also suffers from limited therapeutic options. Despite an aggressive neoadjuvant approach based on cisplatin, doxorubicin, methotrexate and ifosfamide, patients continue to relapse. Fanelli et al. took an in-depth look at cisplatin resistance in osteosarcoma, assessing the value as therapeutic targets of DNA repair-related factors belonging to nucleotide excision repair (NER) or base excision repair (BER) pathways as well as a group kinases. The authors identified NSC130813 (NERI02; F06) and triptolide as valuable agents for overcoming cisplatin resistance, and also confirmed mitogen-activated protein kinase (MAPK) and fibroblast growth factor receptor (FGFR) pathways as novel therapeutic targets in this disease setting.

Another aggressive bone sarcoma and the second most common bone malignancy in young patients is Ewing’s sarcoma, characterized by a specific 11:22 chromosomal translocation that generates the EWS/FLI1 fusion oncogene. This malignancy shows rapid growth and early metastasis. Mancarella et al. identified IGF2BP3 as a promising marker for Ewing sarcoma progression and CD164 and CXCR4 as novel IGF2BP3 downstream functional effectors.

In addition to sarcomas, neuroendocrine neoplasms and endocrine gland tumors represent a large group of heterogeneous rare malignancies. These tumors show a wide variety of clinical presentations and although some progress has been made in recent years in terms of diagnosis and pathology classification, their management and treatment remain challenging. The natural history of these tumors is still poorly understood. For example, pancreatic neuroendocrine tumors (pNETs) account for less than 3% of all pancreatic malignancies. Bocchini et al. provided a comprehensive overview of experimental, prognostic and predictive biomarkers available in clinical practice that can be used to facilitate early diagnosis, estimate prognosis and guide the choice of treatment. Their state-of-the-art paper could be a starting point for further research aimed at improving our understanding and clinical management of this complex disease. Recently, preclinical and retrospective clinical data identified a potential anticancer effect mediated by the oral hypoglycemic agent, metformin, in pNETs. Vernieri et al. studied the impact of the drug on the metabolism of pNET patients and its potential role in the treatment of pNETs. The authors also presented a brief overview of current prospective trials investigating the activity of metformin in combination with standard therapies in this disease setting.

The limited availability of systemic therapeutic options represents a hot topic within the context of metastatic neuroendocrine neoplasia (NEN). Bongiovanni et al. carried out a systematic review and meta-analysis of the efficacy and safety of the tyrosine kinase inhibitor (TKI) pazopanib in patients with metastatic and locally advanced NEN. Their results confirmed the efficacy of the drug in these subgroups, the overall response rate comparable with that of other TKIs and mTOR inhibitors, and furnished a rationale to better understand the role of pazopanib in these malignancies.

Von Hippel-Lindau (VHL) disease is a complex inherited disorder characterized by several types of tumors (including pNETs) arising in multiple organs. The clinical effects of TKIs on VHL disease-related tumors are still largely unknown. Ma et al. retrospectively analyzed the response of VHL patients to TKIs, their results suggesting a potential activity of these inhibitors in this disease setting with a manageable toxicity profile.

Among endocrine tumors, thyroid cancer represents the most frequent malignancy and anaplastic thyroid cancer (ATC) the most aggressive histotype. ATC is characterized by limited therapeutic regimens and poor prognosis. Lin et al. investigated the combination of GSK-J4 and doxorubicin in *in vitro* and *in vivo* anaplastic thyroid cancer models, reporting an activity of the treatment in KRAS-mutant ATC.

Cholangiocarcinoma is a rare and highly fatal malignant tumor of the bile duct, with a poorly understood biological and clinical behavior. Although the tumor has a lower incidence in adolescents and young adults (AYA), this subgroup shows the poorest OS. Feng et al. analyzed three different data sets of AYA cholangiocarcinoma, indentifying ASXL1 and KMT2C as potentially targetable genomic signatures for these patients and providing new insights into this disease.

With regard to the central nervous system, glioblastoma (GBM) represents the most aggressive of all brain tumors. Although prognosis is very poor, a methylated state of the MGMT gene promoter has been shown to predict a better response to temozolomide therapy. Brigliadori et al. identified an intermediate range of methylation (*gray zone*) above the standard cutoff in which the predictive strength of the marker was lost. The authors performed a preliminary assessment on samples belonging to the *gray zone*, confirming the hypothesis of a mismatch between methylation values used for clinical decision making and the variability of the methylation status of each sample. Further research is needed to better define the predictive power of this marker.

An important aspect of the era of multidisciplinary cancer research is that of networking, especially for rare tumors. Melis et al. proposed a network model for clinical and translational research into thymic epithelial tumors. This tool could also be used to implement therapeutic and management facilitate strategies within the more general context of rare tumors.

In conclusion, rare tumors now account for 25% of all cancers. Although these tumors have a low incidence of less than 6 per 100,000 inhabitants, in many cases they have a high prevalence (13), indicating the importance of directing our efforts at promoting interdisciplinary collaborations in care, research and educational areas. Understanding the natural history of these tumors would constitute a substantial breakthrough in preventing, diagnosing earlier and more accurately, and proposing new targeted and interdisciplinary therapeutic approaches.

## Author Contributions

All authors contributed to the article and approved the submitted version.

## Funding

J-YB: NetSARC+ (INCA & DGOS), RHU4 DEPGYN (ANR-18-RHUS-0009)], PIA Institut Convergence Francois Rabelais PLAsCAN (PLASCAN, 17-CONV-0002), LYRICAN (INCA-DGOS-INSERM 12563), la Fondation ARC, InterSARC (INCA), LabEx DEvweCAN (ANR-10-LABX-0061), Ligue de L’Ain contre le Cancer, La Ligue contre le Cancer, EURACAN (EC 739521).

## Conflict of Interest

The authors declare that the research was conducted in the absence of any commercial or financial relationships that could be construed as a potential conflict of interest.
